# Flexural strength, flexural modulus and microhardness of milled vs. fused deposition modeling printed Zirconia; effect of conventional vs. speed sintering

**DOI:** 10.1186/s12903-023-03829-8

**Published:** 2024-01-07

**Authors:** Maher S. Hajjaj, Rana A. A. Alamoudi, Walaa A. Babeer, Waleed Y. Rizg, Ahmad A. Basalah, Saeed J. Alzahrani, Hanin E. Yeslam

**Affiliations:** 1 Department of Restorative Dentistry, Faculty of Dentistry, King Abdulaziz University, Jeddah, Saudi Arabia; 2 Prosthodontic Master Student, Department of Oral and Maxillofacial Rehabilitation, Faculty of Dentistry, King Abdulaziz University, Jeddah, Saudi Arabia; 3 Department of Oral and Maxillofacial Rehabilitation, Faculty of Dentistry, King Abdulaziz University, Jeddah, Saudi Arabia; 4 Department of Pharmaceutics, Faculty of Pharmacy, King Abdulaziz University, Jeddah, 21589 Saudi Arabia; 5 Center of Innovation in Personalized Medicine (CIPM), 3D Bioprinting Unit, King Abdulaziz University, Jeddah, 21589 Saudi Arabia; 6 Mechanical Engineering Department, College of Engineering and Architecture, Umm Al Qura University, Makkah, Saudi Arabia; 7 Advanced Technology Dental Research Laboratory, King Abdulaziz University, P.O. Box 80209, Jeddah, 21589 Saudi Arabia

**Keywords:** Restorative dentistry, Prosthodontics, Zirconia, FDM, Mechanical properties, Sintering cycle, 3D printing, CAD/CAM

## Abstract

**Background:**

Various methods can be used for creating zirconia dental restorations, including 3-dimensional (3D) printing and computer-aided design/ computer-aided manufacturing (CAD/CAM) milling. The fused deposition modeling (FDM) printing method for zirconia presents numerous advantages, albeit research on the mechanical properties of these materials and resultant restorations remains scarce. Such developments are undeniably intriguing and warrant further investigation. The objective of the present study was to evaluate the impact of the sintering firing cycle (Conventional vs. Speed sintering) on the flexural strength, flexural modulus, and Vickers Microhardness of milled vs. FDM printed zirconia.

**Methods:**

A total of 60 bars (2 × 5 × 27 mm) were fabricated for flexural strength testing, along with 40 discs (12 × 1.5 mm) for Vickers microhardness testing. Half of the specimens underwent conventional sintering, while the other half underwent a speed sintering cycle. The flexural strength and modulus were determined by a three-point bending test in a universal testing machine. The microhardness of the specimens was evaluated using a Vickers microhardness tester. Statistical analysis was performed using a two-way ANOVA test with a post-hoc Tukey test (p < 0.05).

**Results:**

CAD/CAM milled zirconia had significantly higher flexural strength and modulus than FDM-printed zirconia. The sintering process did not significantly affect the flexural strength or modulus of milled or FDM-printed zirconia. The milled speed sintering group had significantly higher values in the Vickers microhardness test compared to the other groups.

**Conclusions:**

The mechanical properties of FDM-printed zirconia specimens were not found to be comparable to those of milled zirconia. Speed sintering cycle may produce milled zirconia restorations with similar flexural strength and modulus to conventional sintering, and even higher Vickers Microhardness values.

## Background

Zirconia has been used widely in the dental field to restore mutilated teeth by partial or full coverage prostheses and replace missing teeth by implant supported prostheses due to its high biocompatibility, chemical stability, esthetics and good mechanical properties [1, 2]. In addition, even with more conservative tooth preparation, monolithic zirconia is an opaque material that enables the clinician to mask a metal post or discolored tooth. Moreover, it eliminates the risk of veneering ceramic chipping that is encountered with porcelain fused to metal [3, 4]. A national cross-sectional study of dental practitioners in the United States of America concluded that zirconia materials were the preferred material for the fabrication of indirect dental restorations and crowns on posterior teeth [5].

Since the introduction of dental zirconia, the most common production method has been computer-aided design and computer-aided manufacturing (CAD/CAM), specifically through subtractive manufacturing (SM) technologies (milling). In this method, the intended prosthesis is fabricated by subtracting the material from a prefabricated zirconia block or blank [6, 7]. Milling zirconia can be categorized into two subcategories: soft milling of partially sintered blocks which is then followed by sintering, or hard milling of fully sintered blocks into the final shape and size [8]. There are several advantages of SM including homogenous materials, fast production time [9], and quality control by eliminating some of the conventional laboratory steps such as wax-up, investing and casting [10].

Despite the numerous advantages of milling technologies, there is a significant amount of raw material that is wasted in the process [1]. Also, milling accuracy is limited to reproducing fine details of complex prostheses due to machinery angulation and the size of the milling tools [2]. Moreover, the cost of wear and abrasion of the milling machine and burs [3]. To overcome milling’s drawbacks, additive manufacturing (AM) technology is emerging to address these limitations [4].

AM technologies or Three-Dimensional (3D) printing produces planned objects with incremental layering [5]. 3D printing emerged widely in the dental field due to the ability to print complex prostheses with fine details in a short time and minimal material waste [6]. 3D printed technology is used in dentistry to fabricate diagnostic casts, implant placement guides, provisional materials and night guards [7–9]. 3D printing advancement enables the dental laboratory technicians to print polymers, metal and ceramics [10, 11]. Recently, zirconia materials were fabricated by 3D printing technologies [12, 13].

There are multiple methods of 3D printing available for printing zirconia including Stereolithography (SLA), Direct Light Processing (DLP), Selective Laser Melting (SLM), Selective Laser Sintering (SLS), Fused Deposition Modeling (FDM), and Direct Inkjet Printing (DIP) [14]. SLA and DLP both use deposition liquid-based 3D printed material including photosensitive resin from vat, with the only difference between the two methods being the source of light curing [15, 25]. SLM and SLS both use powder-based 3D printed materials and uses layering techniques. However, SLM uses one-step curing for the full object [16]. DIP uses ceramic suspension deposited from a heated nozzle and each droplet undergoes a phase transition to solid after contacting the surface [17]. FDM works by extruding the 3D-printed material in layering methods after being heated above the melting temperature [14].

Multiple authors aimed to evaluate the mechanical properties of the 3D printed zirconia to milled zirconia. Nakai et al. [18] investigated the flexural strength of three different SLA-printed zirconia to milled zirconia and found comparable results. Abualsaud et al. [19] evaluated the microhardness and flexural strength of SLA 3D printed zirconia with three printing orientations and compared them to milled zirconia. They found that there was no significant difference between milled and 3D printed zirconia in microhardness but flexural strength influenced significantly with the angle of printing. Miura et al. [20] found that the printing orientation influenced the SLA-printed zirconia. Bergler et al. [21] found that flexural strength has no significant difference between milled and 3D printed. In addition, chewing simulation and thermocycling did not influence the flexural strength of the 3D-printed zirconia. Attempt to fabricate zirconia with the FDM method of printing were achieved and the result of the flexural strength showed potential for using this technique [22].

Printing metal and polymers are managed differently than zirconia. Achieving fully dense objects directly with a single step, for metal by printing with SLS or SLM and for polymers with printers using curable resin [23]. The steps of printing zirconia are indirect multiple steps, first printed in green bodies followed by removing the binder material used to shape the zirconia particles (debinding), and end with sintering to obtain the fully dense object [24, 25].

Printing with FDM offers multiple advantages compared to other printing methods such as the low machine fees, simple operation, and reduced cost of printed raw material [22, 26]. However, it should be noted that, up to the authors knowledge, there is a dearth of evidence in the literature regarding the application of 3D printed zirconia restorations produced through FDM techniques [22, 26, 27]. Thus, for FDM-printed zirconia to be clinically applicable in the production of dental restorations and prostheses, its mechanical properties, including flexural strength and microhardness, must be comparable to the milled zirconia. The objectives of this study were to compare the flexural strength, flexural modulus, and Vickers Microhardness of milled zirconia to FDM-printed zirconia. Also, to evaluate the effect of the sintering firing cycle (conventional vs. speed sintering) on these mechanical properties. The first null hypothesis was that there is no significant difference in flexural strength, flexural modulus and Vickers microhardness between milled and FDM-printed zirconia. The second null hypothesis was that the sintering speed cycle (conventional vs. speed sintering) has no significant effect on flexural strength, flexural modulus, and Vickers microhardness of milled and FDM-printed zirconia.

## Methods

### Sample size calculation

G*Power 3.1.9.7 statistical software was used to compute the required sample size. Effect size f was set to 0.5 at significance level of α = 0.05 and power of 0.8, with 4 test groups. The computed total sample size was 42 specimens, which approximately require 10 specimens/group. We used 15 specimens/ group for flexural strength test and 10 specimens/ group for microhardness test.

### Experimental specimens preparation

#### Milled zirconia specimens

30 bars (2 × 5 × 27 mm) for the flexural strength test and 20 discs (12 × 1.5 mm thickness) for the Vickers microhardness test were milled out of pre-sintered zirconia blocks (Ceramill ZI, AMANNGIRRBACH AG., Herrschaftswiesen 1, 6842 Koblach, Austria). Specimens then were polished using silicon carbide abrasive papers with 320-, 400-, 600-, and 1200 grit for 10 s under water cooling (MetaServ 250, Buehler; Illinois, USA). Then, specimens were cleaned with distilled water using an ultrasonic cleaner (PowerSonic 405, Hwashin; Seoul, South Korea), for 5 min.

#### Printed zirconia specimens

3D models of 30 bars (2 × 5 × 27 mm) for flexural strength test and 20 discs (12 × 1.5 mm thickness) for Vickers Microhardness test were generated with the assistance of the CAD package of the Craftware Pro v1.1.4.95 (Craftunique Kft., Budapest, Hungary). The designed models were sliced using the same software and exported into 2D layers in a g-code format to a 3D printing machine. Specimens were printed employing the FDM in the 3D printing process of zirconia structures, using commercially available White Zirconia Zetamix Filament (Nanoe SAS, Ballainvilliers, France). The fabrication of the printed group (green bodies) was conducted using a Craftbot Plus Pro 3D Printer (Craftunique Kft., Budapest, Hungary). Throughout the printing process, the manufacturer’s instructions were meticulously followed to ensure optimal results. Printing temperature was set to 180 °C, and the plate temperature to 40 °C, at a speed of 30 mm/s, and a 0.2 mm layer thickness with a nozzle diameter of 0.6 mm. The FDM-printed specimens were too brittle and could not be polished before sintering.

Before sintering, the green bodies were subjected to a two-stage debinding procedure to remove the thermoplastic binder. The first step was chemical debinding, where the binder was dissolved in an acetone bath in a special holding furnace (Thermo Electoron T6, Thermo Scientific, Langenselbold, Germany) at 40 °C for 3 h. Specimens were then dried for 24 h. The second step was thermal debinding, where the leftover binder was thermally dissolved, specimens were placed in a crucible in a burn-out furnace (Miditherm 100 MP, BEGO, Bremen, Germany) with a heating rate of 60 °C/h from 20 to 500 °C. Table 1 summarizes the compositions and production of materials used in this study.

**Table 1:** Compositions and manufacturers of the materials used in the present study

Material	Production	Composition	Grain size	Manufacturer
Ceramill ZI	Milling	ZrO_2_, HfO_2_, Y_2_O_3_, Al_2_O_3_ and other Oxides	≤ 0.6 μm	AMANNGIRRBACH AG ^a^
Zetamix White Zirconia	FDM	ZrO_2_, Y_2_O_3_, thermoplastic binder	≈ 0.4 μm [28, 29]	NANOE SAS ^b^

^a^ Herrschaftswiesen, Austria

^b^ Ballainvilliers, France

### Sintering

#### Conventional sintering

Half of the milled and FDM-printed specimens (15 bars and 10 discs/group) were subjected to conventional sintering cycle using a sintering furnace (Programat S1 1600, Ivoclar, Schaan, Liechtenstein) according to manufacturer instructions. The heating rate in the first stage was 10 °C/min with a maximum temperature of 900 °C and a holding time of 20 min. In the second stage, the heating rate was 5 °C/min with an end temperature of 1500 °C, a holding time of 120 min, and a cooling rate of 10 °C/min to 300 °C. The total sintering time for the conventional sintering cycle was about 7 h (Table 2).

#### Speed sintering

The remaining 15 bars and 10 discs/group of the milled and FDM-printed specimens were subjected to a speed firing cycle using the same sintering furnace. The heating rate in the first stage was 90 °C/min with a maximum temperature of 900 °C and a holding time of 30 min. The heating rate in the second stage was 60 °C/min with an end temperature of 1500 °C, a holding time of 60 min and a cooling rate of 60 °C/min. The total time for the speed sintering cycle was about 2 h (Table 2).

**Table 2:** Conventional and speed sintering cycles protocols

Sintering Cycle	Start Temp	Heating rate first stage	Max Temp first stage	Holding time	Heating rate second stage	Max Temp second stage	Holding time	Cooling rate	Cooling to
**Conventional Sintering**	Room temp	10 °C/min	900 °C	20 min	5 °C/min	1500 °C	120 min	10 °C/min	300 °C
**Speed** **Sintering**	Room temp	90 °C/min	900 °C	30 min	60 °C/min	1500 °C	60 min	60 °C/min	300 °C

#### Polishing

All specimens were polished according to the following process: using silicon carbide papers with 320-, 400-, 600-, and 1200 grit for 5 min under continuous water cooling (MetaServ 250, Buehler; Illinois, USA). Then, specimens were cleaned with distilled water using an ultrasonic cleaner (PowerSonic 405, Hwashin; Seoul, South Korea), for 5 min.

The CAD/CAM milled specimens were polished before sintering. Therefore, they received no further polishing treatment following sintering. FDM-printed specimens were fully sintered then polished using the above-mentioned procedure. Because these specimens were fully sintered, it took significantly longer time and more effort to reach the fully polished surface.

#### Artificial aging

All specimens were subjected to artificial aging in deionized water baths between 5 and 55 °C using an automatic thermocycling machine for 5000 cycles (SD Mechatronik Thermocycler, JULABO GmbH; Seelbach, Germany). Each cycle took 1 min to be completed. This aging regimen corresponds to 6-months of intraoral simulation [30]. The machine was checked daily to ensure uninterrupted cycles, stable temperature, and adequate water level.

#### Flexural strength test

For the flexural strength test, specimens were tested using a 3-points bending test in a universal testing machine (INSTRON; Norwood, MA, USA) at a cross head speed of 1 mm/min with a distance of 20 mm between the supports. Flexural strength and modulus were measured using Bluehill 3 software (Version 3.24.1496, Instron Worldwide Headquarters, Norwood, MA, USA). The following formula was used to compute the flexural strength (S):

$${\text{S}}=3{\text{FL}}/2{\text{b}}{{\text{d}}^2}$$

Where (S) flexural strength is measured in MPa, (F) load at break or yield is measured in Newtons, (L) specimen span between supports = 20 mm, (b) specimen width = 5 mm, (d) specimen thickness = 2 mm.

The following formula was used to compute the flexural modulus (E) in MPa:

$${\text{E}}={{\text{F}}_1}{{\text{L}}^3}/4{\text{b}}{{\text{d}}^3}{{\text{D}}_1}$$

Where (E) is elastic modulus, (F_1_) deflection force, (L) specimen span between supports = 20 mm, (b) specimen width = 5 mm, (d) specimen thickness = 2 mm, D_1_ deflection at linear region of load-deflection curves.

#### Vickers Microhardness Test

A Vickers hardness tester (HMV microhardness tester, SHIMADZU, Kyoto, Japan) was used to test the specimen for microhardness. Five indentations were created on the surface of each sample using a 9.8 N load. Each indentation lasted for 10 s and was placed at least 1 mm apart from the others. Each indent was measured alone then the mean value was calculated.

The following formula was used to calculate the Vickers hardness number (VHN):

$${\text{HVN}}\,=\,1.8544{\text{ P}}/{{\text{d}}^2}$$

Where P is the applied load (kg), and d is the mean of indentations (mm).

### Statistical analysis

Statistical analysis was performed utilizing JMP 17 Statistical Discovery from SAS software (SAS Campus Drive. Cary, NC, USA). Smirnov-Kolmogorov test was used to assess the normal distribution of the flexural strength, modulus, and microhardness data in each tested group. Data followed a normal distribution (*p* < 0.05). Consequently, Two-way ANOVA was used to evaluate the effect of fabrication technique (milled vs. FDM), sintering cycle (conventional vs. speed), and their interaction on the flexural strength, modulus, and Vickers Microhardness of zirconia followed by post-hoc Tukey test for pair-wise comparison at a significance level of α = 0.05.

## Results

Flexural strength, flexural modulus, and Vickers microhardness for milled vs. FDM printed zirconia were compared in this study. The effect of the sintering cycle (conventional vs. speed sintering) on these mechanical properties was also evaluated. Descriptive statistics and statistical analyses are presented in Table 3.

**Table 3:** Descriptive and statistical analysis of the flexural strength, flexural modulus and microhardness for milled and FDM groups

Groups (Fabrication technique/ sintering cycle)	Flexural Strength (MPa) Mean ± SD	Flexural Modulus (GPa) Mean ± SD	Vickers Microhardness (VHN) Mean ± SD
Milled/ Conventional	1241.22 ± 200.88 ^A^	93.2 ± 3.85 ^A^	1415.62 ± 79.7 ^B^
Milled/ Speed Sintering	1162.8 ± 140.0 ^A^	94.34 ± 5.5 ^A^	1622.1 ± 216.2 ^A^
FDM/ Conventional	257.45 ± 39.8 ^B^	60.85 ± 8.45 ^B^	1329.36 ± 31.68 ^B^
FDM/ Speed Sintering	237.91 ± 52.1 ^B^	58.64 ± 10.9 ^B^	1348.9 ± 62.06 ^B^
**Two-way ANOVA** ***p*** **-value**	< 0.0001*	< 0.0001*	< 0.0001*
**Two-Way ANOVA for independent variables** ***p*** **-value**			
Fabrication technique	< 0.0001*	< 0.0001*	< 0.0001*
Sintering cycle	0.1948	0.7998	0.0053*
**Two-Way ANOVA for independent variables interaction factor** ***p*** **-value**			
Interaction factor (Fabrication*Sintering)	0.4332	0.4128	0.0190*

*Significant at *p* < 0.05

Similar superscript letters within the same category (column) indicate no statistically significant difference (*p* < 0.05)

**Fig. 1:**
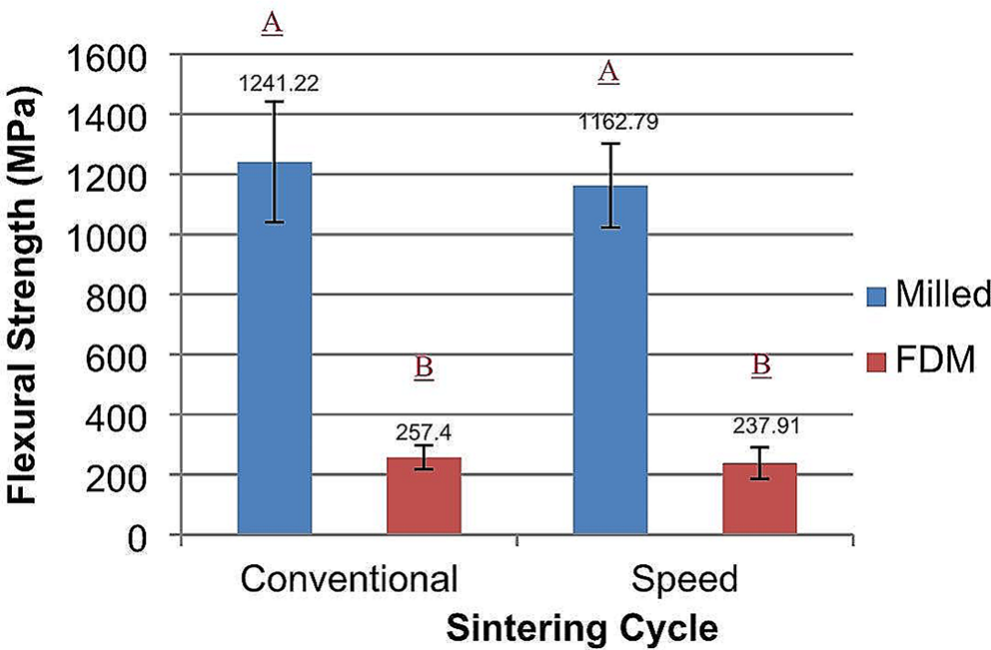
Flexural strength of milled and FDM printed zirconia, the effect of different sintering cycles. Similar letters indicate no statistically significant difference (*p* < 0.05)

For flexural strength data, the two-way ANOVA test showed statistically significant differences between test groups (*p* < 0.0001). Regarding the independent variables and their interaction factor effect, only the fabrication technique had a significant effect on the flexural strength (*p* < 0.0001), but neither the sintering cycle (*p* = 0.1948) nor the interaction factor (*p* = 0.4332) had a significant effect on the flexural strength. Post-hoc Tukey test revealed that there was no significant difference between the conventional and speed-sintered milled zirconia groups (1241.22 ± 200.88 MPa and 1162.8 ± 140.0 MPa) respectively, but they were significantly higher than the FDM printed groups (257.45 ± 39.8 MPa and 237.91 ± 52.1 MPa) (Fig. 1).

**Fig. 2:**
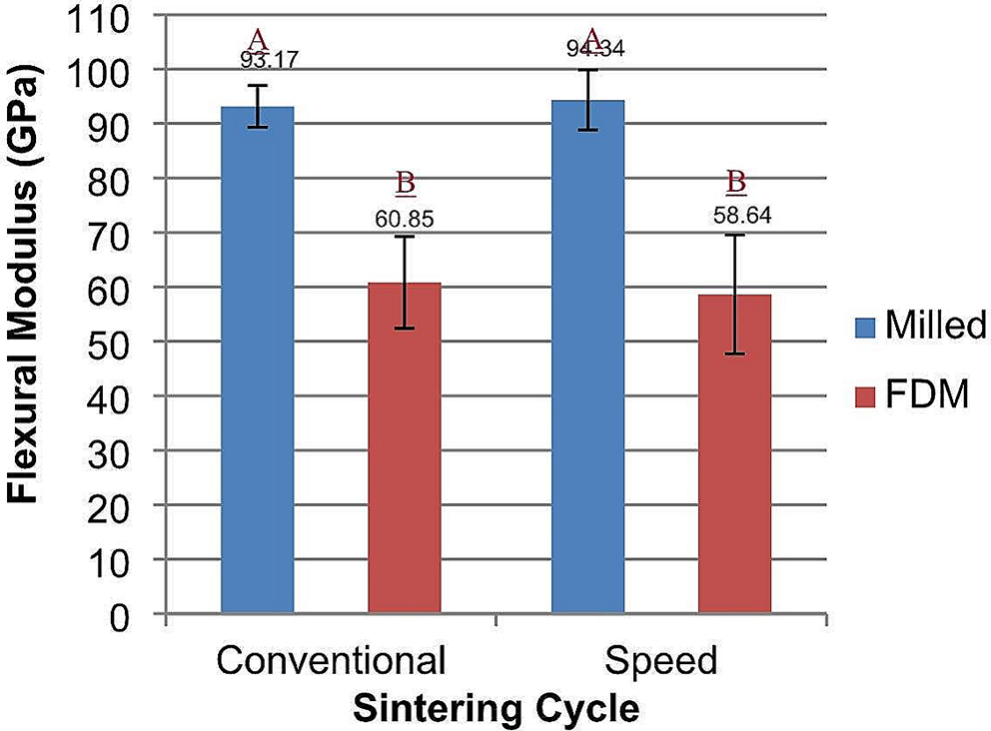
Flexural modulus of milled and FDM printed zirconia, the effect of different sintering cycles. Similar letters indicate no statistically significant difference (p < 0.05)

Similar findings were also reported with flexural modulus data. Two-way ANOVA test showed statistically significant differences between the test groups (*p* < 0.0001). Regarding the independent variables and their interaction factor effect, only the fabrication technique had a significant effect on the flexural strength (*p* < 0.0001), but neither the sintering cycle (*p* = 0.7998) nor the interaction factor (*p* = 0.4128) had a significant effect on the flexural modulus. Post-hoc Tukey test revealed that there was no significant difference between the conventional and speed-sintered milled zirconia groups (93.2 ± 3.85 GPa and 94.34 ± 5.5 GPa) respectively, but they were significantly higher than the FDM printed groups (60.85 ± 8.45 GPa and 58.64 ± 10.9 GPa) (Fig. 2).

**Fig. 3:**
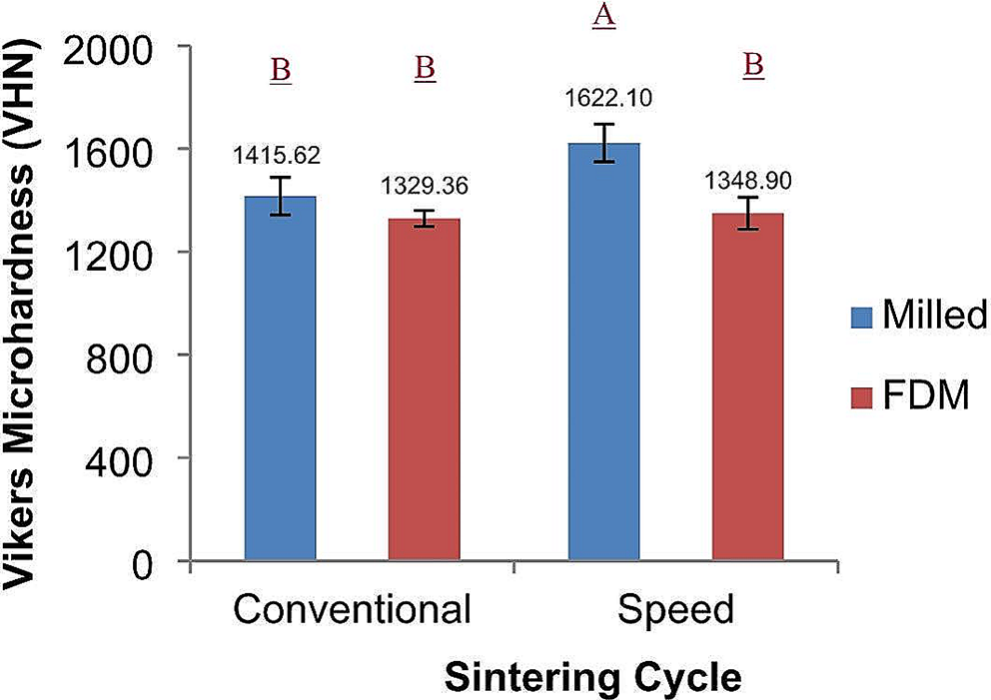
Vickers Microhardness of milled and FDM printed zirconia, the effect of different sintering cycles. Similar letters indicate no statistically significant difference (p < 0.05)

For Vickers microhardness data, the two-way ANOVA test showed statistically significant differences between test groups (*p* < 0.0001). The fabrication technique (*p* = < 0.0001), sintering cycles (*p* = 0.0053) and the interaction factor (*p* = 0.019) had a significant effect on the microhardness. Post-hoc Tukey test revealed that the milled speed sintering group was significantly higher than the rest of the groups, which were not significantly different from one another (Fig. 3).

## Discussion

Additive manufacturing (AM) technologies, also known as 3D printing, have revolutionized the dental industry by offering an efficient and cost-effective way to produce complex dental restorations [31]. Zirconia ceramic restorations are increasingly being fabricated using these technologies [32]. The current study was designed to evaluate two different fabrication techniques for zirconia; subtractive manufacturing by milling and AM by FDM printing method with two different sintering cycles; conventional and speed sintering. Three different mechanical properties: flexural strength, flexural modulus and microhardness were investigated. All specimens were subjected to thermal cycling to simulate a 6-month-long intra oral use [33]. According to the results obtained from this study, the first null hypothesis was rejected because the milled groups had significantly higher flexural strength (*P* < 0.0001), flexural modulus (*P* < 0.0001), and Vickers microhardness values (*P* < 0.0001). The second null hypothesis, which stated that the sintering speed cycle (conventional vs. speed sintering) has no significant effect on flexural strength, flexural modulus, and Vickers microhardness, was also rejected due to the milled speed group having significantly higher microhardness values than the rest of the groups [21, 34–36, 36]. Thermocycling is a method used to quickly simulate intraoral variations in thermal and fatigue resistances that cause mechanical stresses and cracks in restorations that can affect their longevity [33, 36]. To mimic real-life conditions of 6 months of intraoral use, maintain consistency, and reduce variability in the results, all samples underwent the same thermal aging protocol of 5,000 cycles (5–55 °C) [33, 37, 38]. Used in a recent study by Zenthöfer et al., [39] comparing strength of milled and 3D-printed zirconia after different conditioning.

According to ISO 6872 specification, ceramics are classified into five classes based on their intended clinical use [40]. Based on the findings of the current study, FDM-printed zirconia groups, with mean flexural strength less than 300 MPa, would be only indicated for adhesively cemented single-unit anterior or posterior restoration (minimum 100 MPa) (Class 2). For the milled zirconia groups, with a mean flexural strength over 800 MPa, they can be used as monolithic ceramics or substructures of partial- or full-coverage prostheses consisting of four or more units FDPs (Class 5). Bergler et al. [21] found that there was no significant difference between the flexural strength of DLP printed zirconia (855.4 ± 112.6 MPa) and milled zirconia (936.3 ± 255 MPa). Furthermore, the chewing simulation and thermocycling did not negatively impact the flexural strength of the printed zirconia. On the other hand, Revilla-León et al. [41] found significant differences between SLA printed and milled zirconia and mastication simulation significantly influenced the flexural strength. Osman et al. [42] found the flexural strength of DLP-printed zirconia is comparable with the conventional method. The flexural strength of the present study showed the flexural strength of the FDM 3D printed zirconia has one-fifth of the milled zirconia. This significant decrease might relate to the flaws inherited in the printing techniques. The process of fabrication of zirconia by AM involves multiple steps and cracks initiated during the furnace treatment [13]. This mostly happens during the burning out of the polymer binder (debinding) which requires meticulous handling [43]. The values of the present study for flexural strength are lower, which might be due to the production technique, the process of printing, the binder and the zirconia load.

For microhardness, All samples had values within the expected range for microhardness of zirconia (> 1200 VHN) [44]. Abualasaud et al. [19] found no significant difference between milled and SLA-printed zirconia with three different printing orientations. Baysal et al. [45] showed a significant difference between milled and printed zirconia by jetting printing method. In the present study, only milled speed sintering showed higher values and significant differences with the remaining groups.

Conventional zirconia sintering is a process that requires 6–8 h due to the slow heating and cooling rates which are often 5-10^o^ C /min [46]. However, Speed sintering was introduced for a more effective protocol since the heating and cooling rates are much faster and dwell time is significantly shorter from hours to several minutes [47]. In the current study, two sintering protocols were used to compare their influence on the flexural strength, flexural modulus, and microhardness. The result showed no impact of the protocol of sintering on the tested mechanical properties, which is in agreement with multiple previous studies [25, 46–50]. Öztürk and CeliK [51] found that there is a significant difference of heating rate on the flexural strength of zirconia. Furthermore, Upon X-ray diffraction analysis (XDR), they found no difference in the zirconia grain size with different heating rates. Stawarczyk et al. [52] found that an increase in the sintering temperature negatively influences the flexural strength of the materials. In the current study, there was no significant difference in flexural strength between the conventional and speed sintering protocols (P < 0.0001).

All samples had values within the expected range for microhardness of zirconia. This is explained by the fact that the microhardness is affected by the microstructure which is similar in both milled and 3D printed samples. Even though the ability to use a binder in 3D printed ceramic allows the production of various ceramic materials, the process is still time-consuming due to the multiple steps involved in the production process, which is a controversial issue when compared to the rapid production of subtractive manufacturing (milling). One-step printing, which does not involve binder material, is faster. Unfortunately, this process is not yet available for printing dental zirconia restoations [13].

AM technologies offer a significant advantage over traditional SM dental restorative procedures by producing intricate structures with precision while conserving materials [53]. This study involves multiple limitations including its in vitro design, where the specimens were performed in simple material design. It might be more clinical if the specimens were made in real crown shape to evaluate the mechanical properties. Additionally, only FDM was used in this study to 3D print zirconia samples. Recently, Komissarenko et al. reported that digital light processing (DLP) can also produce high-strength, dense zirconia with superior quality [54]. Abualsaud et al. reported the strength of SLA-printed zirconia but was affected by printing orientation [19]. Therefore, further research comparing the mechanical properties of FDM zirconia to SLA-, SLS-, multi-jetting-, and/or DLP-printed zirconia against milled zirconia is recommended. Another limitation is related to the fine details involved in dental production, evaluation of the trueness and precision is an important factor in comparing the different zirconia production methods of 3D printing and milling. An additional limitation includes not performing a topography analysis of the printed materials. Therefore, more research is recommended to evaluate the dynamic mechanical properties of dental FDM zirconia printing is recommended [54]. Given that monolithic crowns have only 45% clinically acceptable color compared to natural teeth, [55] polychromatic zirconia discs/blocks for milling may produce improved esthetic results [32]. Glass veneers are often used to enhance the appearance of zirconia restorations, especially for 3D-printed ones, as they can conceal their inherently layered structure [56]. Recently, Theis et al. [57] suggested that the color of 3D-printed zirconia can be affected by variations in the sintering technique used. Therefore, it is recommended that additional research be conducted to evaluate the mechanical, esthetic properties, and durability of 3D-printed zirconia using FDM in comparison to SLA or DLP methods.

## Conclusions

Within the limitations of this in vitro study, the following conclusions can be drawn:

1. The fabrication technique had a significant effect on the flexural properties of produced zirconia specimens. CAD/CAM milled zirconia had significantly higher flexural strength and modulus than FDM-printed zirconia, therefore it may present a favorable fabrication technique for restorations in high stress situations.
2. FDM-printing may produce zirconia specimens with comparable microhardness values to milled zirconia. Therefore, FDM 3D printing technique might be a viable option for the efficient fabrication of zirconia restorations, but needs more research and improvement to reach clinically acceptable flexural properties.
3. The sintering protocol did not have a negative impact on the mechanical properties of the FDM-printed zirconia in the current study, where speed sintering cycle produced milled zirconia restorations with similar flexural strength and modulus to conventional sintering with even higher Vickers Microhardness values. Therefore, speed sintering may be a more cost-effective and time-saving process for the production of milled zirconia restorations, benefiting both the patient and dental practitioner.

## Data Availability

The data presented in this study are available on request from the corresponding author.

## Abbreviations

^o^ C: Degrees Celsius
2D: Two Dimensional
3D: Three Dimensional
AM: Additive manufacturing
ANOVA: Analysis of variance
CAD/CAM: Computer-aided design/ Computer-assisted manufacturing
DIP: Direct Inkjet Printing
DLP: Direct Light Processing
FDM: Fused Deposition Modeling
GPa: Gega Pascal
s: Seconds
min: Minutes
h: Hours
mm: Millimeter
MPa: Mega Pascal
N: Newtons
SD: Standard deviation
SM: Subtractive manufacturing
SLA: Stereolithography
SLM: Selective Laser Melting
SLS: Selective Laser Sintering
VHN: Vickers Hardness Number
